# Voice handicap and voice-related quality of life in COVID-19 patients

**DOI:** 10.1016/j.bjorl.2024.101437

**Published:** 2024-04-25

**Authors:** Lourival Mendes Bueno, Hugo Valter Lisboa Ramos, Claudiney Cândido Costa, Wilder Alves, Leandro Castro Velasco, Noemi Grigoleto De Biase

**Affiliations:** a Programa Interinstitucional de Pós-Graduação do Centro de Reabilitação e Readaptação Dr. Henrique Santillo (CRER), São Paulo, SP, Brazil; b Universidade Federal de São Paulo, Escola Paulista de Medicina (UNIFESP-EPM), São Paulo, SP, Brazil; c Centro de Reabilitação e Readaptação Dr. Henrique Santillo Dr. Henrique Santillo (CRER), Goiânia, GO, Brazil; d Pontifícia Universidade Católica de São Paulo (PUC-SP), São Paulo, SP, Brazil

**Keywords:** Voice, Voice quality, Lung injury, COVID-19

## Abstract

**Objectives:**

Identifying voice handicap and voice-related quality of life in patients presenting pulmonary impairment associated with COVID-19 infection, comparing pulmonary parameters between these patients and individuals in the control group, as well as correlating pulmonary parameters to self-assessment questionnaires (IDV-10 and QVV).

**Methods:**

Thirty-five (35) patients presenting pulmonary impairment with COVID-19 infection were herein selected and compared to 35 individuals who were not affected by COVID-19 infection. Two self-assessment questionnaires were applied (vocal handicap index and voice quality of life protocol). Maximum phonation time Forced Expiratory Pressure (PEF) and Forced Inspiratory Pressure (PIF) were measured and videolaryngoscopy was performed.

**Results:**

There was statistically significant difference in scores recorded in voice self-assessment questionnaires (IDV-10 and QVV), Expiratory Pressure (PEF) and Forced Inspiratory Pressure (PIF) between patients with pulmonary impairment associated with COVID-19 infection and those in the control group. Correlation between PEF/PIF and scores recorded in voice self-assessment questionnaires was also observed.

**Conclusion:**

Pulmonary impairment associated with COVID-19 infection has worsened voice handicap and voice-related quality of life in the assessed patients, as well as reduced their forced expiratory and inspiratory pressure in comparison to the control group.

**Level of evidence:**

4.

## Highlights

• Lung impairment due to COVID-19 increases the voice handicap index score.
• The voice-related quality of life score is reduced in these patients.
• There is a correlation between pulmonary function and the changes in voice.
• Video laryngoscopy did not identify any laryngeal changes in these patients.

## Introduction

COVID-19 infection caused by the SARS-COV2 virus has a wide spectrum of clinical manifestations that range from asymptomatic patients to fatal cases.^1^, ^2^ It is clinically featured by the acute phase, which corresponds to the initial illness, whose duration can range from days to four weeks, on average.^1^, ^2^ Post COVID-19 syndrome, also known as long COVID-19, corresponds to persistent symptoms lasting for more than 4 weeks.^3^, ^4^

Pulmonary impairment associated with COVID-19 infection is caused by direct viral action in patients’ alveolar epithelium and endothelial cells, in addition to virus-independent mechanisms, such as hyperinflammation and thrombotic phenomena in individuals’ pulmonary vasculature. Its clinical symptoms comprise dyspnea and cough, as well as acute respiratory failure in severe cases.^5^, ^6^, ^7^, ^8^ Some patients may develop persistent respiratory symptoms, mainly dyspnea, whose prevalence is observed in 42%‒66% of infected individuals.^5^, ^6^, ^7^, ^8^

Voice-related complaints are described in the literature at incidence rates ranging from 20% to 40% in patients affected by COVID-19 infection.^8^, ^9^, ^10^ Several hypotheses have been put forward to explain the pathophysiology of vocal impairments caused by this infection.^11^, ^12^, ^13^ Human vocal folds are associated with high expression of angiotensin-converting enzyme 2, which is COVID-19 receptor. These findings could explain the inflammation process observed in these patients’ vocal folds.^14^, ^15^, ^16^

Another hypothesis lies on the post-viral vagal neuropathy, which compromises both the sensory and motor branches of the vagus nerve, and leads to symptoms, such as dysphonia, vocal fatigue, dysphagia, globus and laryngospasm.^17^, ^18^, ^19^

In addition to the herein described pathophysiology types, pulmonary impairment caused by COVID-19 infection can reduce subglottal pressure and, consequently, reduce phonation efficiency.^19^, ^20^, ^21^

Orotracheal intubation can also damage patients’ larynx and, consequently, their voice. Granuloma, vocal fold paralysis and subglottic stenosis were the main injuries observed after mechanical ventilation.^20^, ^21^

Although several studies have evidenced vocal changes at the acute COVID-19 infection phase, only few studies focused on assessing vocal handicap and voice-related quality of life of patients with pulmonary impairment.

The aims of the current study were to identify vocal handicap and changes in voice-related quality of life in patients with pulmonary impairment associated with COVID-19 infection, to compare lung parameters between patients with lung impairment associated with COVID-19 infection and individuals in the control group, as well as to investigate whether there is correlation between lung parameters and self-assessment questionnaires.

## Methods

Cross-sectional, descriptive study conducted with patients presenting pulmonary impairment associated with COVID-19 infection, from August 2021 to January 2022. It was carried out at the respiratory physiotherapy outpatient clinic of a tertiary hospital.

This research was approved via *Plataforma Brasil*, under protocol nº 5.168.099. All participants have signed the Free and Informed Consent Term (FICT).

Study sample was formed by volunteers who were split into pulmonary impairment and control groups. The pulmonary impairment group comprised patients presenting pulmonary impairment associated with COVID-19 infection, who had their condition confirmed through RT-PCR and chest tomography, and who required follow-up based on respiratory physiotherapy. Data about this group were compared to those of the control group, which was formed by non-smokers with no history of COVID-19 infection, and whose age and sex matched those of the pulmonary impairment group. Individuals in the control group were recruited among individuals who accompanied the patients,It was done in the same hospital where study-group data were collected and where volunteers’ were invited to participate in the study.

Male and female patients with pulmonary impairment associated with COVID-19 infection, in the age group 18–65 years, who required respiratory physiotherapy and who agreed to participate in the research, were included in it after signing the Free and Informed Consent Term. Smokers, patients with history of either chronic obstructive pulmonary disease or other laryngeal changes, as well as patients subjected to intubation during hospitalization due to COVID-19 infection were excluded from the sample.

Participants were subjected to the application of two questionnaires: Voice Handicap Index 10 (VHI-10) and Voice-Related Quality of Life (V-RQoL) protocol. VHI-10 (Voice Handcap Index) is a self-assessment questionnaire used to measure vocal changes’ influence on individuals’ quality of life. It comprises 10 questions and score 40 means maximum disadvantage. Costa et al. have validated VHI-10’s Portuguese version.^22^

V-RQoL, in its turn, assesses voice quality of life through 10 questions about 3 different aspects of individuals, namely: physical, social-emotional and global domains. Higher V-RQoL scores are related to better quality of life. Gasparini et al. validated V-RQoL protocol’s Portuguese version.^23^

In addition to applying the aforementioned questionnaires, the current study also assessed individuals’ Maximum Phonation Time (MPT). Participants were instructed to inhale through their nose and to emit phoneme /a/ right away, for as long as possible. Three measurements were taken, and their highest values were taken into consideration.

Moreover, participants had lung parameters, such as maximal inspiratory and expiratory pressures, measured with the aid of analog manovacuometer. They were asked to inhale deeply and to exhale in the aforementioned device, with as much force as possible, to measure maximum forced expiratory pressure. On the other hand, participants were asked to inhale deeply into the device to measure forced inspiratory pressure. Three measurements were taken, and their highest values were expressed in centimeters of H_2_O and taken into consideration.

Videolaryngoscopy was only performed in the pulmonary impairment group. This procedure was carried out in the respiratory physiotherapy room, with the aid of Toshiba microcamera equipped with rigid-optic 70° (Storz brand), under topical oropharyngeal anesthesia with 10% lidocaine. Images were analyzed by three otolaryngologists with expertise in laryngology, as well as blindly edited and randomized, with 20% case repetitions. The aforementioned physicians observed vocal folds’ mobility and “arching”, as well as incidence of injuries, stenosis and synechia.

Results in the current research, except for videolaryngoscopy results, were analyzed by comparing data recorded for the pulmonary impairment and control groups. Variables were initially analyzed through Shapiro-Wilk test to determine whether their central tendency measures (mode, mean and median) presented normal distribution.

Non-parametric Mann-Whitney test was used to compare patients in the pulmonary impairment group to individuals in the control group. Statistical analyses were carried out in Minitab® software v. 19, in R® software v. 4.2 and in Stata software v. 16.0, at 5% significance level. Non-parametric Spearman test was used to analyze correlations among variables in the pulmonary impairment group. It was done to check whether there was any correlation between pulmonary parameters and voice self-assessment questionnaires.

Fleiss Kappa test (for three, or more, examiners) was used to analyze inter-examiner agreement among results, whereas Cohen Kappa test was applied to analyze intra-examiner results’ reliability. All herein performed tests presented similar interpretations ranging from 0% to 100% reliability and/or agreement. Statistical analyses were carried out in R software v. 4.2, in association with RStudio compatible to this version, at 5% significance level.

## Results

Seventy (70) volunteers were included in the sample, as well as split into pulmonary impairment and control groups. Both groups comprised 35 participants, each. The age and sex of participants in the control group matched those of the pulmonary impairment group.

The mean age of all 35 patients in the pulmonary impairment group was 47.7 years (±10.72), whereas the mean age of participants in the control group was 44.8 years (±9.73). Table 1, Table 2 also show the mean and standard deviation of the main results recorded for the pulmonary impairment and control groups.

**Table 1:** Distribution of the group with COVID-19 based on individuals’ age, VHI-10, V-RQoL, MPT, FEP, FIP and COVID-19 duration.

Variations	Age (years)	VHI-10	V-RQoL	MPT(s)	FEP	FIP	COVID-19 duration (days)
***N* (general framework)**	35	35	35	35	35	35	35
Mean	47.7	5.97	81.9	13.9	98.1	81.4	152
Standard deviation	10.7	5.55	16.5	7.39	21.7	25.5	101
***N* (Male)**	22	22	22	22	22	22	22
Mean	48.77	4.86	85.91	16.41	106.36	93.18	153.64
Standard deviation	10.69	5.99	16.05	7.57	15.90	21.02	103.29
***N* (Female)**	13	13	13	13	13	13	13
Mean	45.85	7.85	75.19	9.58	84.23	61.54	148.46
Standard deviation	10.95	4.32	15.59	4.77	23.62	19.51	100.40

VHI-10, Voice Handicap Index-10; V-RQoL, Voice-Related Quality of Life; MPT, Maximum Phonation Time; FEP, Forced Expiratory Pressure; FIP, Forced Inspiratory Pressure.

**Table 2:** Distribution of the control group based on individuals’ age, VHI-10, V-RQoL, MPT, FEP and FIP.

Variations	Age (years)	VHI-10	V-RQoL	MPT(s)	FEP	FIP
***N* (general framework)**	35	35	35	35	35	35
Mean	44.8	0.086	96.9	17.4	109	96.9
Standard deviation	9.73	0.284	15.2	8.68	12.9	18.4
***N* (Male)**	19	19	19	19	19	19
Mean	46.21	0.00	99.47	21.70	116.32	108.42
Standard deviation	8.41	0.00	1.05	9.22	7.61	12.14
***N* (Female)**	16	16	16	16	16	16
Mean	43.13	0.19	93.91	12.39	100.63	83.13
Standard deviation	11.14	0.40	22.40	4.33	12.89	14.93

VHI-10, Voice Handicap Index-10; V-RQoL, Voice-Related Quality of Life; MPT, Maximum Phonation Time; FEP, Forced Expiratory Pressure; FIP, Forced Inspiratory Pressure.

The herein analyzed data presented abnormal distribution. Therefore, non-parametric tests were used to compare data recorded for the two groups (Table 3).

**Table 3:** Mann Whitney Test used to compare median values between the pulmonary impairment and control groups (male sex, female sex, and general framework).

Variables	Male sex		Female sex		General framework	
	Median	*p*-Value	Median	*p*-Value	Median	*p*-Value
VHI-10 (control)	0	0.05	0	0.0001	0	0.0001
VHI-10 (pulmonary impairment )	2		6		4	
V-RQoL (control)	100	0.0001	100	0.0001	100	0.0001
V-RQoL (pulmonary impairment )	91.25		80		85	
MPT (control)	22	0.04	11.45	0.1	16	0.07
MPT (pulmonary impairment )	16.05		8		12.4	
FEP (control)	120	0.02	100	0.04	110	0.04
FEP (pulmonary impairment )	110		80		100	
FIP (control)	110	0.02	80	0.003	100	0.01
FIP (pulmonary impairment )	100		60		80	

VHI-10, Voice Handicap Index-10; V-RQoL, Voice-Related Quality of Life; MPT, Maximum Phonation Time; FEP, Forced Expiratory Pressure; FIP, Forced Inspiratory Pressure.

Table 3 presents the results of comparisons between the pulmonary impairment and control groups. All parameters analyzed for participants belonging to the male sex have shown statistically significant differences between groups. However, MPT did not show statistically significant difference between groups in the general framework and in participants belonging to the female sex.

Table 4 presents results of correlations carried out in the pulmonary impairment group. There was significant correlation between pulmonary parameters and voice self-assessment questionnaires.

**Table 4:** Spearman Correlation Test applied to the pulmonary impairment group.

Correlation between variables	Spearman’s correlation	95% CI	*p*-Value	Sample
MPT and VHI-10	−0.378	(−0.639; −0.039)	0.025	General
FEP and VHI-10	−0.389	(−0.647; −0.051)	0.021	General
FIP and VHI-10	−0.467	(−0.702; −0.141)	0.005	General
FEP and V-RQoL	0.367	(0.027; 0.631)	0.03	General
FIP and V-RQoL	0.367	(0.027; 0.631)	0.03	General

VHI-10, Voice Handicap Index-10; V-RQoL, Voice-Related Quality of Life; FEP, Forced Expiratory Pressure; FIP, Forced Inspiratory Pressure.

Videolaringoscopy was performed in patients with pulmonary impairment associated with COVID-19 infection. Results’ agreement between examiners ranged from 85.7% to 94.3%. They are shown in Table 5.

**Table 5:** Agreement between examiners (inter-agreement).

Variables	Cohen *p*-value	Agreement
Normal mobility	0.001	94.30%
Lack of “arching”	0.0001	91.40%
Lack of injuries	0.0005	88.60%
Lack of stenosis	0.0001	91.40%
Lack of synechia	0.01	85.70%

Intra-examiner reliability ranged from 90% to 100%. Results are shown in Table 6, Table 7, Table 8.

**Table 6:** Intra-examiner reliability, based on Cohen Kappa test applied to examiner 1.

Examiner 1		
Variables	Cohen *p*-value	Reliability
Normal mobility	0.05	90%
Lack of “arching”	0.0009	100%
Lack of injuries	0.0009	100%
Lack of stenosis	0.0009	100%
Lack of synechia	0.0009	100%

**Table 7:** Intra-examiner reliability, based on Cohen Kappa test applied to examiner 2.

Examiner 2		
Variables	Cohen *p*-value	Reliability
Normal mobility	0.0001	100%
Lack of “arching”	0.001	90.90%
Lack of injuries	0.001	100%
Lack of stenosis	0.001	100%
Lack of synechia	0.05	90.90%

**Table 8:** Intra-examiner reliability, based on Cohen Kappa test applied to examiner 3.

Examiner 3		
Variables	Cohen *p*-value	Reliability
Normal mobility	0.001	100%
Lack of “arching”	0.001	100%
Lack of injuries	0.001	100%
Lack of stenosis	0.001	100%
Lack of synechia	0.001	100%

## Discussion

The current study assessed voice handicap and voice-related quality of life in patients with pulmonary impairment associated with COVID-19 infection. Two self-assessment questionnaires (VHI-10 and V-RQoL), as well as Maximum Phonation Time (MPT), Forced Expiratory Pressure (FEP), Forced Inspiratory Pressure (FIP) and Videolaryngoscopy were used in the assessment process. Unlike other studies available in the literature, the herein analyzed sample only included patients with pulmonary impairment associated with COVID-19 infection and excluded those who were intubated at this disease’s acute phase.

Results observed for the pulmonary impairment group have shown statistically significant difference in VHI-10 and QVV questionnaires, as well as in PEF and PIF, in comparison to the control group. Higher score was observed for VHI-10 (5.97 ± 5.5) and lower score was observed for V-RQoL (81.9 ± 16.5) between the pulmonary impairment and control groups. Patients’ self-assessment has evidenced worsened voice handicap and low voice-related quality of life. Forced expiratory and inspiratory pressure values were lower in the pulmonary impairment group (98.1 ± 21.7 and 81.4 ± 25.5, respectively) and it evidenced reduced pulmonary function in this group in comparison to the control group. Videolaryngoscopy examinations were only performed in the pulmonary impairment group ‒ results came out normal and presented inter-examiner agreement ranging from 85.7% to 94.3%.

The pulmonary impairment group has also shown negative correlation between self-assessment questionnaires, such as VHI-10 and V-RQoL, and FEP/FIP. These findings have suggested that reduced lung function in patients with pulmonary impairment associated with COVID-19 infection may be linked to the worsened voice handicap and voice-related quality of life observed for this group.

Gölaç et al. compared 40 patients with COVID-19 infection to 40 healthy individuals. The VHI-10 score (2.65 ± 2.73) recorded for the COVID-19 group was higher than that observed for the control group.^24^ Tahir et al. assessed 228 individuals (138 individuals with COVID-19 infection and 90 healthy individuals) and observed higher VHI-10 score (7.31 ± 6.67) in the COVID-19 group.^25^ Shah et al. assessed 57 patients with COVID-19 infection, and they recorded VHI-10 score (11) higher than the one observed for the control group.^26^ Arias et al. assessed 21 patients with COVID-19 and also observed higher IDV-10 score in these patients than in the control group.^27^

Bouldin et al. followed-up 470 patients (split into 2 different groups: hospitalized and non-hospitalized in the acute disease phase) for 12 months and observed higher VHI-10 score (4.9 ± 1.4) in the hospitalized group.^28^ The current findings meet those reported by Golac et al., Tahir et al., Arias et al. and Bouldin et al., who observed slight increase in VHI-10 score. Patients with pulmonary impairment associated with COVID-19 infection have also recorded V-RQoL score lower than that of the control group. This finding was also shared by Gölaç et al.

Shah et al. observed VHI-10 score higher than the one reported in the current study. This finding can be explained by the inclusion of patients subjected to both intubation and tracheostomy procedures in their study.

Forced expiratory and inspiratory pressures were lower in patients with pulmonary impairment associated with COVID-19 infection than in the control group. Krueger et al. assessed lung function in 257 patients presenting mean COVID-19 duration of 112 days and observed reduced FEP and FIP in 24% and 9% of patients, respectively.^29^

Few studies in the literature performed videolaryngoscopy-based assessment in patients with COVID-19 infection. However, these studies included individuals subjected to intubation or tracheostomy procedure.^30^ Thus, the current study was the only one applying videolaryngoscopy in patients with pulmonary impairment associated with COVID-19 infection who were neither intubated nor tracheostomized.

The COVID-19 pandemic has challenged healthcare systems, worldwide. In addition to the straight impact of this disease, its consequences are a significant public health issue. Therefore, further studies must be carried out to help better understanding damages caused by COVID-19 infection to patients’ voices and their impact on these individuals’ quality of life, as well as to assess vaccination effects on reducing these symptoms.

## Conclusion

Patients in the pulmonary impairment group recorded increased voice handicap index score, decreased voice-related quality of life scores based on the questionnaire, as well as reduced lung function parameters (FEP and FIP) in comparison to the control group.

There was negative correlation between VHI-10 questionnaire and FEP/FIP, as well as positive correlation between V-RQoL questionnaire and FEP/FIP. This finding suggested that changes observed in the self-assessment questionnaires had pulmonary origin.

## Funding

Author himself.

## Conflicts of interest

The authors declare no conflicts of interest.
